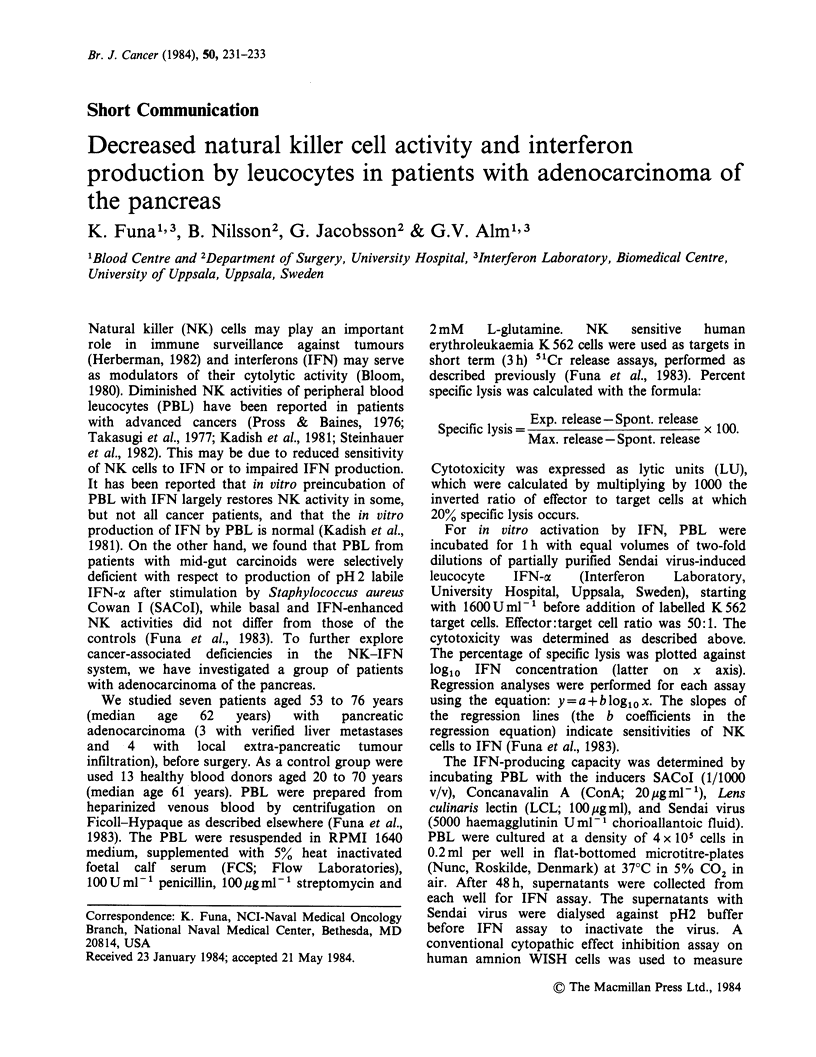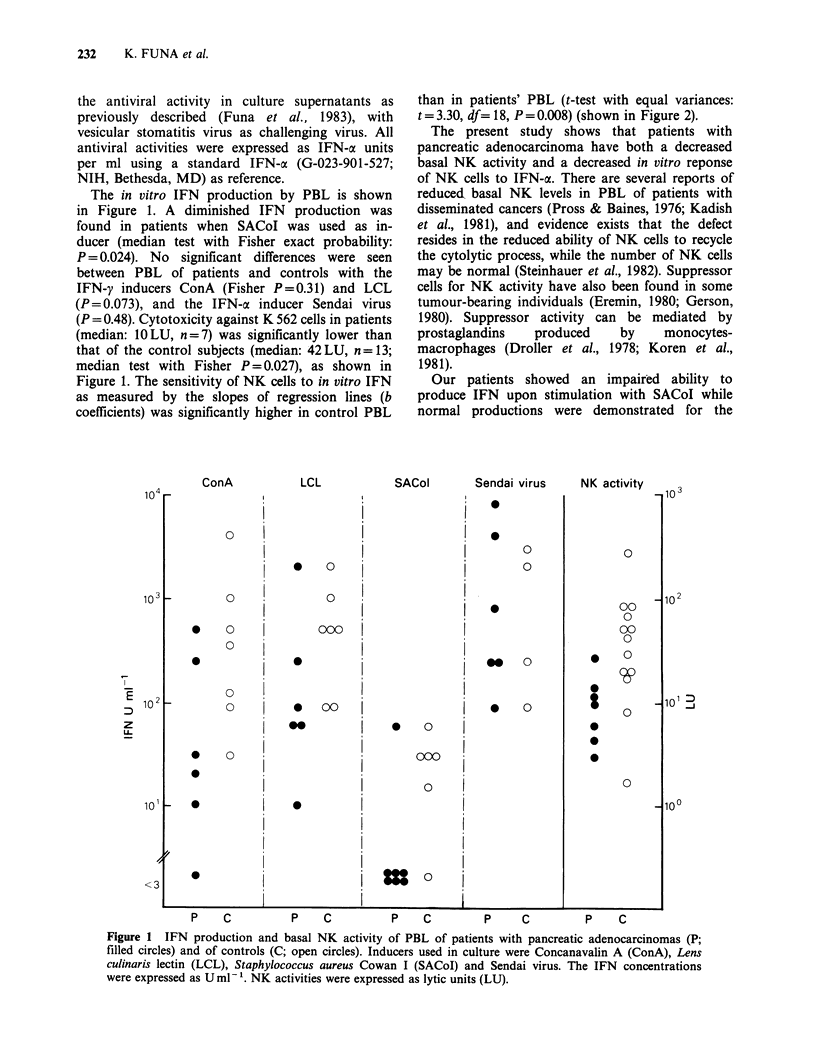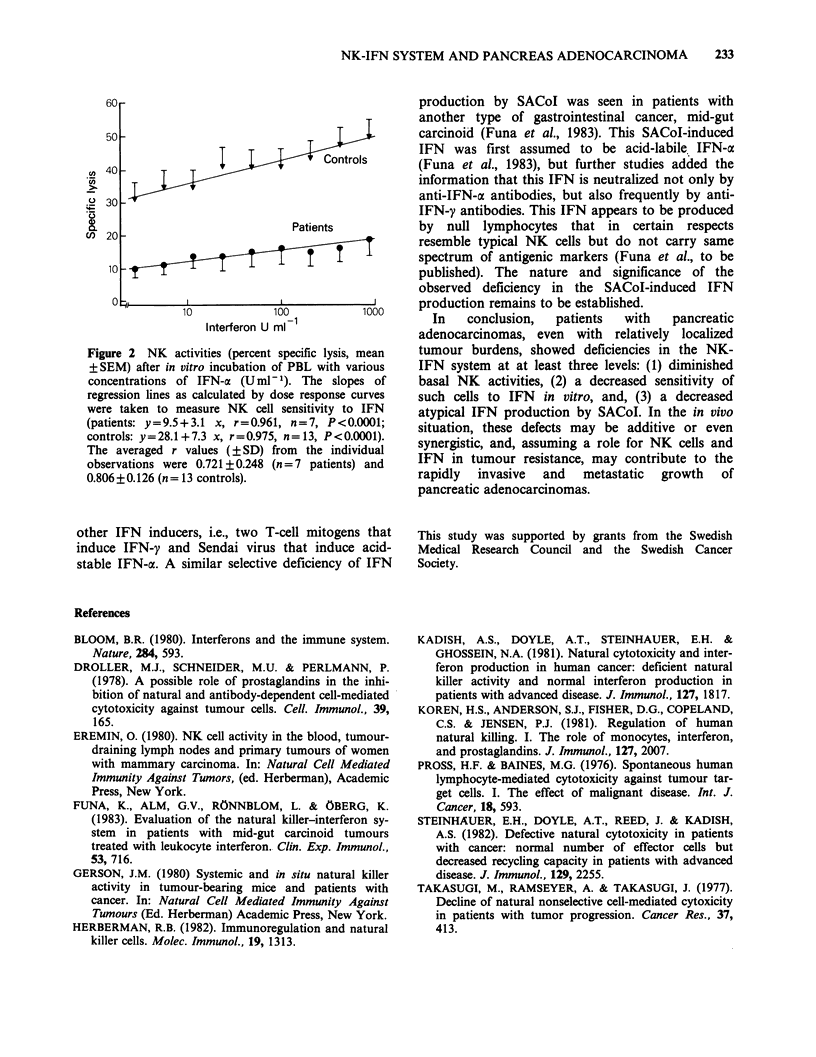# Decreased natural killer cell activity and interferon production by leucocytes in patients with adenocarcinoma of the pancreas.

**DOI:** 10.1038/bjc.1984.168

**Published:** 1984-08

**Authors:** K. Funa, B. Nilsson, G. Jacobsson, G. V. Alm


*Br. J. Cancer* (1984), **50**, 231–233

**Short Communication**

### Decreased natural killer cell activity and interferon production by leucocytes in patients with adenocarcinoma of the pancreas

K. Funa[1,3], B. Nilsson[2], G. Jacobsson[2] & G.V. Alm[1,3]

[1]*Blood Centre and* [2]*Department of Surgery, University Hospital,* [3]*Interferon Laboratory, Biomedical Centre, University of Uppsala, Uppsala, Sweden*

Natural killer (NK) cells may play an important role in immune surveillance against tumours (Herberman, 1982) and interferons (IFN) may serve as modulators of their cytolytic activity (Bloom, 1980). Diminished NK activities of peripheral blood leucocytes (PBL) have been reported in patients with advanced cancers (Pross & Baines, 1976; Takasugi *et al.*, 1977; Kadish *et al.*, 1981; Steinhauer *et al.*, 1982). This may be due to reduced sensitivity of NK cells to IFN or to impaired IFN production. It has been reported that *in vitro* preincubation of PBL with IFN largely restores NK activity in some, but not all cancer patients, and that the *in vitro* production of IFN by PBL is normal (Kadish *et al.*, 1981). On the other hand, we found that PBL from patients with mid-gut carcinoids were selectively deficient with respect to production of pH 2 labile IFN-$\alpha$ after stimulation by *Staphylococcus aureus* Cowan I (SACoI), while basal and IFN-enhanced NK activities did not differ from those of the controls (Funa *et al.*, 1983). To further explore cancer-associated deficiencies in the NK–IFN system, we have investigated a group of patients with adenocarcinoma of the pancreas.

We studied seven patients aged 53 to 76 years (median age 62 years) with pancreatic adenocarcinoma (3 with verified liver metastases and 4 with local extra-pancreatic tumour infiltration), before surgery. As a control group were used 13 healthy blood donors aged 20 to 70 years (median age 61 years). PBL were prepared from heparinized venous blood by centrifugation on Ficoll–Hypaque as described elsewhere (Funa *et al.*, 1983). The PBL were resuspended in RPMI 1640 medium, supplemented with 5% heat inactivated foetal calf serum (FCS; Flow Laboratories), 100 U ml$^{-1}$ penicillin, 100 $\mu$g ml$^{-1}$ streptomycin and 2 mM L-glutamine. NK sensitive human erythroleukaemia K 562 cells were used as targets in short term (3 h) $^{51}$Cr release assays, performed as described previously (Funa *et al.*, 1983). Percent specific lysis was calculated with the formula:

$$\text{Specific lysis} = \frac{\text{Exp. release} - \text{Spont. release}}{\text{Max. release} - \text{Spont. release}} \times 100.$$

Cytotoxicity was expressed as lytic units (LU), which were calculated by multiplying by 1000 the inverted ratio of effector to target cells at which 20% specific lysis occurs.

For *in vitro* activation by IFN, PBL were incubated for 1 h with equal volumes of two-fold dilutions of partially purified Sendai virus-induced leucocyte IFN-$\alpha$ (Interferon Laboratory, University Hospital, Uppsala, Sweden), starting with 1600 U ml$^{-1}$ before addition of labelled K 562 target cells. Effector:target cell ratio was 50:1. The cytotoxicity was determined as described above. The percentage of specific lysis was plotted against $\log_{10}$ IFN concentration (latter on $x$ axis). Regression analyses were performed for each assay using the equation: $y = a + b\log_{10} x$. The slopes of the regression lines (the $b$ coefficients in the regression equation) indicate sensitivities of NK cells to IFN (Funa *et al.*, 1983).

The IFN-producing capacity was determined by incubating PBL with the inducers SACoI (1/1000 v/v), Concanavalin A (ConA; 20 $\mu$g ml$^{-1}$), *Lens culinaris* lectin (LCL; 100 $\mu$g ml), and Sendai virus (5000 haemagglutinin U ml$^{-1}$ chorioallantoic fluid). PBL were cultured at a density of $4 \times 10^5$ cells in 0.2 ml per well in flat-bottomed microtitre-plates (Nunc, Roskilde, Denmark) at 37°C in 5% $CO_2$ in air. After 48 h, supernatants were collected from each well for IFN assay. The supernatants with Sendai virus were dialysed against pH2 buffer before IFN assay to inactivate the virus. A conventional cytopathic effect inhibition assay on human amnion WISH cells was used to measure

Correspondence: K. Funa, NCI-Naval Medical Oncology Branch, National Naval Medical Center, Bethesda, MD 20814, USA
Received 23 January 1984; accepted 21 May 1984.

© The Macmillan Press Ltd., 1984

the antiviral activity in culture supernatants as previously described (Funa *et al.*, 1983), with vesicular stomatitis virus as challenging virus. All antiviral activities were expressed as IFN-α units per ml using a standard IFN-α (G-023-901-527; NIH, Bethesda, MD) as reference.

The *in vitro* IFN production by PBL is shown in Figure 1. A diminished IFN production was found in patients when SACoI was used as inducer (median test with Fisher exact probability: $P=0.024$). No significant differences were seen between PBL of patients and controls with the IFN-γ inducers ConA (Fisher $P=0.31$) and LCL ($P=0.073$), and the IFN-α inducer Sendai virus ($P=0.48$). Cytotoxicity against K 562 cells in patients (median: 10 LU, $n=7$) was significantly lower than that of the control subjects (median: 42 LU, $n=13$; median test with Fisher $P=0.027$), as shown in Figure 1. The sensitivity of NK cells to *in vitro* IFN as measured by the slopes of regression lines (*b* coefficients) was significantly higher in control PBL than in patients' PBL (*t*-test with equal variances: $t=3.30$, $df=18$, $P=0.008$) (shown in Figure 2).

The present study shows that patients with pancreatic adenocarcinoma have both a decreased basal NK activity and a decreased *in vitro* reponse of NK cells to IFN-α. There are several reports of reduced basal NK levels in PBL of patients with disseminated cancers (Pross & Baines, 1976; Kadish *et al.*, 1981), and evidence exists that the defect resides in the reduced ability of NK cells to recycle the cytolytic process, while the number of NK cells may be normal (Steinhauer *et al.*, 1982). Suppressor cells for NK activity have also been found in some tumour-bearing individuals (Eremin, 1980; Gerson, 1980). Suppressor activity can be mediated by prostaglandins produced by monocytes-macrophages (Droller *et al.*, 1978; Koren *et al.*, 1981).

Our patients showed an impaired ability to produce IFN upon stimulation with SACoI while normal productions were demonstrated for the

**Figure 1** IFN production and basal NK activity of PBL of patients with pancreatic adenocarcinomas (P; filled circles) and of controls (C; open circles). Inducers used in culture were Concanavalin A (ConA), *Lens culinaris* lectin (LCL), *Staphylococcus aureus* Cowan I (SACoI) and Sendai virus. The IFN concentrations were expressed as U ml$^{-1}$. NK activities were expressed as lytic units (LU).

**Figure 2** NK activities (percent specific lysis, mean ±SEM) after *in vitro* incubation of PBL with various concentrations of IFN-α (U ml$^{-1}$). The slopes of regression lines as calculated by dose response curves were taken to measure NK cell sensitivity to IFN (patients: $y=9.5+3.1\ x$, $r=0.961$, $n=7$, $P<0.0001$; controls: $y=28.1+7.3\ x$, $r=0.975$, $n=13$, $P<0.0001$). The averaged $r$ values (±SD) from the individual observations were 0.721±0.248 ($n=7$ patients) and 0.806±0.126 ($n=13$ controls).

other IFN inducers, i.e., two T-cell mitogens that induce IFN-γ and Sendai virus that induce acid-stable IFN-α. A similar selective deficiency of IFN production by SACoI was seen in patients with another type of gastrointestinal cancer, mid-gut carcinoid (Funa *et al.*, 1983). This SACoI-induced IFN was first assumed to be acid-labile IFN-α (Funa *et al.*, 1983), but further studies added the information that this IFN is neutralized not only by anti-IFN-α antibodies, but also frequently by anti-IFN-γ antibodies. This IFN appears to be produced by null lymphocytes that in certain respects resemble typical NK cells but do not carry same spectrum of antigenic markers (Funa *et al.*, to be published). The nature and significance of the observed deficiency in the SACoI-induced IFN production remains to be established.

In conclusion, patients with pancreatic adenocarcinomas, even with relatively localized tumour burdens, showed deficiencies in the NK-IFN system at at least three levels: (1) diminished basal NK activities, (2) a decreased sensitivity of such cells to IFN *in vitro*, and, (3) a decreased atypical IFN production by SACoI. In the *in vivo* situation, these defects may be additive or even synergistic, and, assuming a role for NK cells and IFN in tumour resistance, may contribute to the rapidly invasive and metastatic growth of pancreatic adenocarcinomas.

This study was supported by grants from the Swedish Medical Research Council and the Swedish Cancer Society.

### References

BLOOM, B.R. (1980). Interferons and the immune system. *Nature*, **284**, 593.

DROLLER, M.J., SCHNEIDER, M.U. & PERLMANN, P. (1978). A possible role of prostaglandins in the inhibition of natural and antibody-dependent cell-mediated cytotoxicity against tumour cells. *Cell. Immunol.*, **39**, 165.

EREMIN, O. (1980). NK cell activity in the blood, tumour-draining lymph nodes and primary tumours of women with mammary carcinoma. In: *Natural Cell Mediated Immunity Against Tumors*, (ed. Herberman), Academic Press, New York.

FUNA, K., ALM, G.V., RÖNNBLOM, L. & ÖBERG, K. (1983). Evaluation of the natural killer–interferon system in patients with mid-gut carcinoid tumours treated with leukocyte interferon. *Clin. Exp. Immunol.*, **53**, 716.

GERSON, J.M. (1980) Systemic and *in situ* natural killer activity in tumour-bearing mice and patients with cancer. In: *Natural Cell Mediated Immunity Against Tumours* (Ed. Herberman) Academic Press, New York.

HERBERMAN, R.B. (1982). Immunoregulation and natural killer cells. *Molec. Immunol.*, **19**, 1313.

KADISH, A.S., DOYLE, A.T., STEINHAUER, E.H. & GHOSSEIN, N.A. (1981). Natural cytotoxicity and interferon production in human cancer: deficient natural killer activity and normal interferon production in patients with advanced disease. *J. Immunol.*, **127**, 1817.

KOREN, H.S., ANDERSON, S.J., FISHER, D.G., COPELAND, C.S. & JENSEN, P.J. (1981). Regulation of human natural killing. I. The role of monocytes, interferon, and prostaglandins. *J. Immunol.*, **127**, 2007.

PROSS, H.F. & BAINES, M.G. (1976). Spontaneous human lymphocyte-mediated cytotoxicity against tumour target cells. I. The effect of malignant disease. *Int. J. Cancer*, **18**, 593.

STEINHAUER, E.H., DOYLE, A.T., REED, J. & KADISH, A.S. (1982). Defective natural cytotoxicity in patients with cancer: normal number of effector cells but decreased recycling capacity in patients with advanced disease. *J. Immunol.*, **129**, 2255.

TAKASUGI, M., RAMSEYER, A. & TAKASUGI, J. (1977). Decline of natural nonselective cell-mediated cytoxicity in patients with tumor progression. *Cancer Res.*, **37**, 413.